# Unraveling the interplay of stress, cognitive failure, and academic self-efficacy among postgraduate nursing candidates: a mediational path analysis

**DOI:** 10.1186/s12912-025-03630-8

**Published:** 2025-07-26

**Authors:** Mona Metwally El-Sayed, Eman Sameh AbdElhay, Bothaina Hussein Hassan, Samah Mohamed Taha

**Affiliations:** 1 Faculty of Nursing, Alexandria University, Alexandria, Egypt; 2 Faculty of Nursing, Mansoura University, Mansoura, Egypt

**Keywords:** Self-efficacy, Latent class analysis, Cognition, Stress, Students, Nursing

## Abstract

**Background:**

Cognitive failure, the inability to perform everyday mental tasks, can be exacerbated by stress, particularly in demanding academic environments such as graduate nursing education. This study examined the relationships between cognitive failure, perceived stress, and academic self-efficacy among postgraduate nursing students.

**Methods:**

A cross-sectional, correlational study was conducted using stratified sampling to recruit 268 postgraduate nursing students from two Egyptian universities. Data were collected through individual structured interviews using validated Arabic versions of the Cognitive Failure Questionnaire (CFQ), Perceived Stress Scale (PSS-10), and Sherer’s General Self-Efficacy Scale (SGSES). Pearson’s correlation assessed the relationships among variables. Independent *t*-tests and one-way ANOVA were used to compare mean scores across demographic groups. Multiple linear regression identified predictors of self-efficacy. Finally, path analysis using AMOS was conducted to test the mediating effect of cognitive failure on the relationship between stress and academic self-efficacy.

**Results:**

Females reported higher cognitive failures than males (M = 53.20 vs. 38.78, *p* < 0.001). Cognitive failure was positively correlated with stress (*r* = 0.181, *p* = 0.003) and negatively correlated with self-efficacy (*r* = − 0.241, *p* < 0.001). Stress also showed a negative correlation with self-efficacy (*r* = − 0.207, *p* = 0.001). Multiple regression revealed that both cognitive failure (β = − 0.170, *p* = 0.001) and perceived stress (β = − 0.483, *p* = 0.005) were significant predictors of reduced self-efficacy, accounting for 7.9% of the variance (*R²* = 0.079, *F* = 12.435, *p* < 0.001). Path analysis confirmed that cognitive failure partially mediated the relationship between stress and self-efficacy (indirect effect = − 0.025, *p* < 0.001; model fit: CFI = 0.918, RMSEA = 0.093).

**Conclusion:**

The findings underscore the mediating role of cognitive failure in the relationship between perceived stress and academic self-efficacy. Interventions focused on reducing stress and enhancing cognitive performance may effectively bolster academic self-efficacy and success among graduate nursing students.

**A clinical trial number:**

Not applicable.

## Introduction

Nursing education is inherently stressful due to the rigorous academic demands combined with high-pressure clinical environments. Nursing students frequently encounter substantial stress arising from the simultaneous responsibilities of mastering theoretical knowledge and applying skills during clinical placements [1, 2]. Stressors in nursing education vary according to the academic year, workload intensity, and clinical setting, with senior students typically reporting higher stress levels compared to their junior counterparts due to increased clinical responsibilities [3, 4].

Significant research identifies several primary stressors, including academic workload, performance expectations in clinical settings, insufficient coping strategies, and environmental factors [5, 6]. Labrague et al. (2018) further highlighted that stress negatively affects nursing students’ physiological and psychological well-being, impairing their learning capabilities and clinical practice performance [7]. Moreover, the transition to online learning amid the COVID-19 pandemic intensified stress levels, exacerbating pre-existing concerns related to academic success [8, 9].

Stress significantly influences cognitive performance, especially regarding attention and memory, which are crucial in nursing contexts characterized by frequent critical decision-making [10–12]. Cognitive failure, brief disruptions in attention or memory lasting between 0.5 and 15 s, pose considerable risks in clinical settings, potentially compromising patient care and safety [13]. Moreover, the prevalence of stress among Egyptian medical students is notably high, with a significant portion experiencing severe stress, which can exacerbate cognitive failure and hinder academic performance [14]. Studies underline stress and depression as substantial predictors of these failures among nursing students [15, 16].

Academic self-efficacy, defined as an individual’s belief in their ability to accomplish academic tasks, is closely linked to cognitive functioning. Diminished cognitive performance due to stress typically corresponds with reduced academic self-efficacy, adversely impacting educational outcomes and emotional well-being [17]. Higher academic self-efficacy correlates with improved academic satisfaction, enhanced coping mechanisms, and better management of stress-related challenges [18]. Furthermore, self-efficacy serves as a protective factor, moderating the adverse effects of stress by bolstering students’ perceived competence and confidence [17].

The unique cultural and educational background of Egyptian nursing graduate students significantly influences the relationship between stress, academic self-efficacy, and cognitive failure. Egyptian nursing students face distinct challenges that shape these relationships, including the need for substantial academic motivation and social support to navigate their educational journey effectively [19]. The cultural context in Egypt emphasizes the importance of social networks and support systems, which are crucial for enhancing intrinsic motivation and academic success [20]. This is consistent with findings from other studies that highlight the role of self-efficacy in managing stress and improving academic performance among nursing students [20].

While numerous studies independently address cognitive failure, stress, and self-efficacy within academic environments, integrated research examining the complex relationships among these variables, particularly in postgraduate nursing education contexts, is limited. Existing literature predominantly emphasizes either stress or self-efficacy separately, neglecting a comprehensive exploration of their interplay with cognitive failure [21]. Specifically, graduate nursing education in Egypt, influenced by unique cultural, educational, and psychological dimensions, lacks sufficient empirical exploration.

Addressing this critical gap, the present study aims to explore the relationship between perceived stress, academic self-efficacy, and cognitive failure among postgraduate nursing students in Egypt. Understanding these dynamics will provide valuable insights for educational practices, student support systems, and interventions designed to enhance academic performance and psychological well-being in nursing education contexts.

### Research questions

#### Q_1_

What are the levels of perceived stress, cognitive failure, and self-efficacy among postgraduate students?

#### Q_2_

What is the relationship between perceived stress and cognitive failure among postgraduate nursing candidates?

#### Q_3_

What is the relationship between academic self-efficacy and cognitive failure among postgraduate nursing candidates?

#### Q_4_

What is the direct and indirect relationship between cognitive failure, perceived stress, and self-efficacy among postgraduate students?

#### Q_5_

Are there significant differences in these relationships based on demographic factors such as age, gender, marital status, or academic degree?

### Research hypotheses

#### H_1_

There is a significant positive relationship between perceived stress and cognitive failure among postgraduate nursing candidates.

#### H_2_

There is a significant negative relationship between cognitive failure and academic self-efficacy among postgraduate nursing candidates.

#### H_3_

**Fig. 1:**
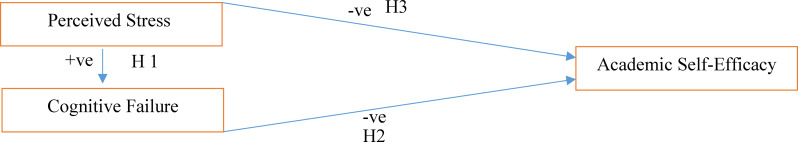
Study conceptual model

There is a significant negative relationship between perceived stress and academic self-efficacy among postgraduate nursing candidates (Fig. 1).

## Methods

### Research design

The current study utilized a cross-sectional descriptive correlational design to examine the relationship between cognitive failure, perceived stress, and self-efficacy among graduate candidates who adhered to the STROBE guidelines checklist [22]. The data were collected from October to the end of December 2024.

### Setting

The study was conducted at the Colleges of Nursing of El-Mansoura and Alexandria Universities in Egypt. This college encompasses nine academic departments: Medical-Surgical Nursing, Critical Care Nursing, Pediatric Nursing, Obstetrics and Gynecological Nursing, Nursing Administration, Nursing Education, Community Health Nursing, Gerontological Nursing, Psychiatric, and Mental Health Nursing. It operates under the Ministry of Higher Education. The faculty offers a range of academic programs for national and international students, including a baccalaureate degree for undergraduate students, diploma certificates, and master’s and doctoral degrees for graduate students. Both the undergraduate and graduate programs utilize a credit hour system. The diploma program spans two semesters, while the master’s program is structured over four semesters and includes the completion of a master’s thesis. The doctoral program consists of six semesters, culminating in the preparation of a doctoral dissertation. Each graduate degree focuses on one of the nursing specialties listed above.

### Target participants

The study focused on participants enrolled in postgraduate programs at the College of Nursing at El-Mansoura and Alexandria Universities for the 2024–2025 academic year. The eligibility criteria were being Egyptian and enrolled in one of the graduate nursing programs, which included diploma, master’s, and doctoral degrees in nursing. Additionally, voluntary participation was a prerequisite for inclusion. Participants with prior-existing psychiatric conditions, such as Major Depressive Disorder, Bipolar I or II Disorder, Generalized Anxiety Disorder, or any other psychiatric conditions, were excluded from the study, particularly those who were actively receiving pharmacological treatment or undergoing therapy for these disorders. These exclusion criteria were rigorously applied to maintain the homogeneity of the participants and minimize the influence of confounding factors.

### Sampling and recruitment

**Fig. 2:**
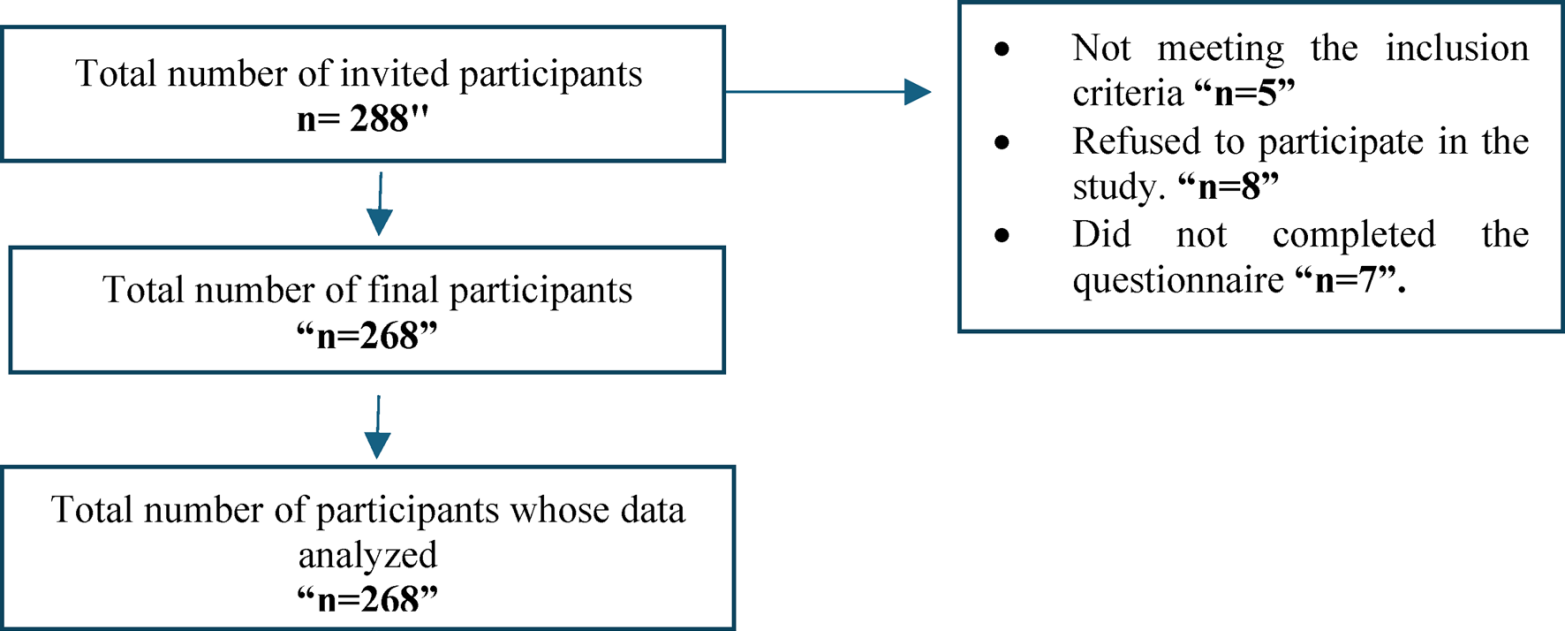
Participants’ recruitment flowchart

After obtaining ethical approval and the necessary permissions from the deans of the two nursing colleges, a list of registered postgraduate nursing students was acquired from the postgraduate affairs office. Using this list as a sampling frame, a stratified sampling technique was employed to ensure representation from different postgraduate nursing programs. A total of 288 candidates were invited to participate in the study. Out of these, 8 candidates declined to take part, and 5 were found to be ineligible. Additionally, 7 participants withdrew from the study without completing the questionnaires. This contributed to a final sample size of 268 participants, achieving a response rate of 93% and a dropout rate of 7%. These 268 participants constituted the core for the subsequent analysis (see Fig. 2).

### Sample size estimation

The required sample was estimated using the following calculation =$\:\:\frac{\left(Z1-\:a/2\right).p(1-p)}{d2}$. The value of Z1 − α/2 is 1.96 for a 5% type I error (*p* < 0.05) [23]. P represents the anticipated proportion in the population derived from previous research, while d denotes the absolute error or precision, d = 0.06 [19, 24]). =$\:\:\frac{\left(1.96)2\right).0.5\text{*}1-0.5)}{\left(0.066\right)2}$ =$\:\:\frac{0.9604}{0.004356}$ =220.5=223, Considering these elements, a sample size of 223 postgraduate candidates was calculated, plus an additional 20% for non-responses. Consequently, 268 participants were incorporated into the study.

### Measurements of interest

The data for this study were collected using the following measurements:

### Participant demographic data

This survey addresses the demographic traits of the participants, including age, gender, marital status, place of residence, and living arrangements, as well as the level of the postgraduate educational program in which they are enrolled and their prior academic background.

### Cognitive failure questionnaire (CFQ)

This questionnaire measured cognitive slips in daily life, such as memory failure and misperceptions, by Broadbent et al. (1982) [25]. It features 25 items rated from 0 (Never) to 4 (Very often), resulting in total scores ranging from 0 to 100, where higher scores indicate more frequent cognitive failure. The CFQ demonstrates strong reliability, with Cronbach’s alpha values between 0.86 and 0.92, and is widely used in clinical and research settings [13, 26, 27]. It has shown good construct validity, with four factors explaining 66.9% of the variance and favorable fit indices (CFI = 0.93, RMSEA = 0.091) [28]. The scale underwent translation into Arabic, followed by back-translation into English by two qualified bilingual experts to ensure conceptual and linguistic equivalence. The Arabic version demonstrated robust psychometric properties. Exploratory factor analysis (EFA) with varimax rotation yielded factor loadings ranging from 0.67 to 0.88, accounting for 79.41% of the total variance. The Kaiser-Meyer-Olkin (KMO) value was 0.950, indicating excellent suitability for factor analysis. All items from the original version were retained. Additionally, the scale showed good internal consistency, with a Cronbach’s alpha of 0.78.

### Perceived stress scale (PSS-10)

It is a self-reported scale designed to gauge how unpredictable, unmanageable, and overburdened individuals felt over the past month [29]. Each response is rated on a five-point Likert-type scale, ranging from 0, indicating “never,” to 4, indicating “very often.” The scale has four statements worded negatively and scored in reverse. The cumulative score is derived by aggregating all item responses, with possible scores ranging from 0 to 40; higher scores reflect a heightened perception of stress. The scale has revealed good internal consistency, with a Cronbach’s alpha of 0.81 [30]. Furthermore, the Arabic PSS-10 version also demonstrated moderate reliability, with a Spearman’s correlation coefficient of 0.74 reported by Chaaya et al. (2010) [31]. This version was used in this study and demonstrated good internal consistency, with a Cronbach’s alpha value of 0.77.

### Sherer’s general self-efficacy scale (SGSES)

Sherer (1982) developed the general self-efficacy scale [32]. It has 17 items; 11 are reversed to avoid response bias. Each item is evaluated on a five-point Likert-type scale that ranges from (1), which signifies strong disagreement, to (5), which indicates strong agreement. The aggregate score ranged from 17 to 85, with higher scores denoting greater self-efficacy. The scale demonstrated commendable internal consistency, as evidenced by Cronbach’s alpha of 0.84 and a correlation coefficient of 0.86 [33]. In this study, the scale was first translated into Arabic. Subsequently, it underwent back-translation into English by two independent bilingual experts to ensure semantic and conceptual equivalence. To evaluate the psychometric properties of the Arabic version, both exploratory factor analysis (EFA) and confirmatory factor analysis (CFA) were conducted. The Kaiser-Meyer-Olkin (KMO) measure of sampling adequacy was exceptionally high at 0.96, indicating the data’s suitability for factor analysis. EFA revealed a robust factor structure, with the 17 items explaining 66.32% of the total variance, and factor loadings ranging from 0.74 to 0.88, indicating strong item contributions. CFA confirmed the model’s adequacy, producing acceptable fit indices: χ²/df = 3.15, RMSEA = 0.007, CFI = 0.931, and TLI = 0.925. The internal consistency of the Arabic version was excellent, as reflected by a Cronbach’s alpha coefficient of 0.88.

### Procedure

#### Ethical approval

The study adhered to the principles outlined in the Declaration of Helsinki, ensuring ethical integrity throughout the research process. Official permissions were secured from the relevant authorities at El-Mansoura University’s College of Nursing and the Research Ethics Committee (REC). Before participation, informed written consent was obtained from all subjects, who were comprehensively informed about the study’s significance and objectives. They were assured that their involvement was entirely voluntary, allowing them to withdraw at any point without consequence. The Research Ethics Committee also approved access to participants’ medical records solely for the purpose of verifying eligibility. All data were handled confidentially and in accordance with institutional privacy guidelines, and anonymity was safeguarded.

#### Validity of the measures

A panel of five experts assessed the face validity of the measures used in the study. They evaluated each item for its relevance, appropriateness, and clarity. The feedback from the experts confirmed that the items were suitable and easily comprehensible, indicating that the measures effectively captured the intended constructs. A confirmatory factor analysis (CFA) was performed to ensure content validity further after translation to Arabic. This statistical method is employed to validate the factor structure of a set of observed variables, confirming that the measurement model accurately represents the underlying constructs being assessed.

#### Preliminary study

A pilot study involving 25 participants was conducted before the primary research to evaluate the clarity and applicability of the CFS, PSS-10, and SGSES. This phase aimed to identify potential obstacles to data collection. Results showed that 22% of participants had mild cognitive impairment, 46% had moderate impairment, and 32% had severe impairment, providing insights into the effectiveness of the measurement tools and areas that needed attention before the main study. Those participants were not included in the primary survey.

#### Data collection

To ensure adherence to the exclusion criteria, a review of participants’ medical records was conducted solely to confirm the absence of any diagnosed psychiatric disorders. The REC approved this review, and strict confidentiality protocols were adhered to in order to protect participants’ privacy. Data collection was conducted through individual, structured interviews with trained researchers who were independent of the participants’ academic instruction or evaluation. These researchers were qualified in structured interviewing techniques and received specific training to ensure standardized and unbiased data collection.

Each interview lasted approximately 10 to 15 min and took place in a private setting, such as a vacant classroom or clinical skills lab, to ensure participant comfort and confidentiality. Informed written consent was obtained from each participant prior to the interview. Participation was entirely voluntary, and students were informed they could withdraw at any point without penalty. No incentives were offered. All responses were handled confidentially, and the research team had no administrative or evaluative authority over the students. The collected data were reviewed for completeness and accuracy by the investigators, ensuring reliability and fostering a respectful, trustworthy environment throughout the data collection process.

### Data analysis

Data analysis was executed using IBM SPSS Statistics software (version 26.0) [34]. After entering the data, a thorough review and verification process was conducted to ensure its accuracy. The normality of the distribution for quantitative variables was assessed using the Kolmogorov-Smirnov (KS) test and the Shapiro-Wilk (SW) test. Cronbach’s alpha was calculated to evaluate the internal consistency of the research tools, thereby showing their reliability. The Kaiser-Meyer-Olkin (KMO) coefficient and Bartlett’s test of sphericity were employed to assess the content validity of the translated measures, specifically the Cognitive Failure Scale (CFS) and the Sherer’s General Self-Efficacy Scale (SGSES). A one-way ANOVA was utilized for statistical comparisons among more than two groups. In contrast, the Student’s t-test was done to compare two groups of normally distributed quantitative variables. Descriptive statistics for CFS, PSS-10, and SGSES were summarized utilizing means, standard deviations, and frequencies or percentages for categorical variables. Pearson’s correlation coefficient was used to evaluate the strength of relationships between two quantitative variables that were normally distributed, as well as to determine the direction of these relationships. Additionally, a multiple linear regression analysis was conducted to verify how cognitive failure and perceived stress, as independent variables, influenced self-efficacy as the dependent variable. Finally, path analysis using AMOS was conducted to test the mediating effect of cognitive failure on the relationship between stress and academic self-efficacy. All results were deemed statistically significant at the 5% level (*p* < 0.05) and a more stringent level of significance (*p* < 0.001).

## Results

Table 1 shows the demographic characteristics of the 268 postgraduate nursing students who participated in the study. In terms of age, 34.32% were 25 years or younger, 47.76% were between 26 and 30 years old, and 17.91% were aged 35 years or older. The majority of participants were female (70.52%), while males accounted for 29.47%. Regarding marital status, 54.47% were married, 39.55% were single, 5.22% were divorced, and 0.71% were widowed. Educationally, 46.28% were enrolled in a master’s program, 27.98% in a doctoral program, and 25.74% were diploma students. Concerning pre-faculty education, 69.40% of participants had completed secondary school, whereas 30.59% were graduates of a technical institute of nursing.

**Table 1:** Demographic characteristics of the participants

Variable	Categories		
		NO	%
Age (in years)	25-	92	34.32
	30-	128	47.76
	35+	48	17.91
Gender	Male	79	29.47
	Female	189	70.52
Marital Status	Single	106	39.55
	Married	146	54.47
	Widowed	2	0.71
	Divorced	14	5.22
Level of Education	Diploma	69	25.74
	Master	124	46.28
	Doctorate	75	27.98
Pre-Faculty Education	Secondary School	186	69.40
	Technical Institute of Nursing	82	30.59

M: Mean; SD: Standard deviation

Table 2 indicates that age had no significant impact on CFQ, PSS, or SGSES scores, with F-values of 0.540, 0.803, and 0.587, and p-values greater than 0.05. However, gender differences were significant; males had a mean CFQ score of 38.78 compared to females at 53.20 (t = 2.666, *p* = 0.000), indicating higher cognitive failure in females. For perceived stress, males averaged 19.05 and females 21.60; however, this difference was not significant (t = 1.681, *p* = 0.215). Marital status significantly affected all measures, with singles reporting the highest CFQ score (58.78); significant differences were noted in CFQ (F = 4.101, *p* = 0.000), PSS (F = 3.874, *p* = 0.002), and SGSES (F = 3.866, *p* = 0.000). Education level showed no significant differences between Master’s and Doctorate holders for CFQ (t = 0.462, *p* = 0.256) or PSS (t = 0.288, *p* = 0.111), but trends in self-efficacy were similar. Finally, no significant differences in CFQ scores were found between secondary school and technical institute graduates (t = 0.150, *p* = 0.303), although the latter reported higher perceived stress (t = 2.478, *p* = 0.002).

**Table 2:** The relationship between participants’ demographic characteristics and study variables (*N* = 268)

Variable	Categories			CFQ		PSS		SGSES	
		NO	%	M	SD	M	SD	M	SD
Age (in years)	25-	92	34.32	51.85	16.44	21.68	4.88	61.48	9.63
	30-	128	47.76	49.93	14.49	20.48	3.77	59.86	10.16
	35+	48	17.91	54.80	16.95	21.81	3.77	62.75	7.25
F (value) Test of sig.				F (0.540) *p* = 0.241		F (0.803) *p* = 0.231		F (0.587) *p* = 0.163	
Gender	Male	79	29.47	38.78	14.58	19.05	4.23	62.56	9.33
	Female	189	70.52	53.20	15.56	21.6	4.35	61.15	9.32
t (value) Test of sig.				t (2.666)** *p* = 0.000		t (1.681) *p* = 0.215		t (0.185) *p* = 0.365	
Marital Status	Single	106	39.55	58.78	15.18	23.25	4.38	58.91	8.37
	Married	146	54.47	50.28	15.62	20.92	4.23	61.59	9.26
	Widowed	2	0.71	33.50	9.19	16.66	3.54	77.00	4.23
	Divorced	14	5.22	39.11	12.18	17.25	3.99	72.35	5.43
F (value) Test of sig.				F (4.101)* *p* = 0.000		F (3.874)* *p* = 0.002		F (3.866)* *p* = 0.000	
Level of Education	Diploma	69	25.74	51.24	15.32	20.14	3.26	59.94	9.23
	Master	124	46.28	52.49	16.48	21.47	4.68	61.41	10.36
	Doctorate	75	27.98	50.97	15.24	21.21	3.92	61.20	8.62
t (value) Test of sig.				F (2.462) *p* = 0.256		F (3.288) *p* = 0.111		F (5.012) *p* = 0.231	
Pre-Faculty Education	Secondary School	186	69.40	51.73	15.70	22.13	3.88	61.36	10.37
	Technical Institute of Nursing	82	30.59	52.24	16.65	19.9	4.96	61.12	6.83
t (value) Test of sig.				t (0.150) *p* = 0.303		t (2.478)* *p* = 0.002*		t (0.016) *p* = 0.171	

t: t-test F: ANOVA *:Statistically significant at *p* ≤ 0.05 **: Statistically significant at *p* ≤ 0.01

M: Mean; SD: Standard Deviation

CFQ: Cognitive Failure Questionnaire

PSS: Perceived Stress Scale

SGSES: Sherer’s General Self-Efficacy Scale

Table 3 presents participants’ mean scores and standard deviations for cognitive failure, perceived stress, and self-efficacy. The CFQ scores ranged from a minimum of 15 to a maximum of 82, with a mean score of 51.90 (SD = 15.95), indicating a moderate level of cognitive failure within the sample. The PSS had scores ranging from 8 to 40, with a mean of 25.21 (SD = 4.38), suggesting that participants experienced moderate perceived stress. Lastly, SGSES scores varied from 40 to 81, with a mean score of 61.28 (SD = 9.28), reflecting a relatively high level of self-efficacy among participants.

**Table 3:** The mean self-efficacy scores, cognitive lapses, and stress (*n* = 268)

Variables	Min.– Max.	M (SD)
CFQ	15–82	51.90 (15.95)
PSS	8–40	25.21 (4.38)
SGSES	40–81	61.28 (9.28)

M: Mean; SD: Standard Deviation

CFQ: Cognitive Failure Questionnaire

PSS: Perceived Stress Scale

SGSES: Sherer’s General Self-Efficacy Scale

Table 4 displays a correlation analysis of participants’ CFQ, PSS, and SGSES. It reveals a significant positive correlation between cognitive failure and perceived stress (*r* = 0.497, *p* = 0.000), indicating that greater cognitive failure is associated with increased perceived stress. Conversely, cognitive failure is negatively correlated with self-efficacy (*r* = -0.448, *p* = 0.000), suggesting that greater cognitive failure is associated with lower self-efficacy. Additionally, stress is negatively correlated with self-efficacy (*r* = -0.207, *p* = 0.001), indicating that as stress levels rise, self-efficacy tends to decline.

**Table 4:** The correlation coefficient between self-efficacy, cognitive lapses, and stress among the participants (*n* = 268)

Variables		CFQ	PSS	SGSES
CFQ	*r* *p*			
PSS	r p	0.181** 0.003**		
SGSES	r p	− 0.241** < 0.001**	-0.207** 0.001**	

r: the Pearson correlation coefficient ^**^: Statistically significant at *p* ≤ 0.01

CFQ: Cognitive Failure Questionnaire

PSS: Perceived Stress Scale

SGSES: Sherer’s General Self-Efficacy Scale

Table 5 summarizes a multiple regression analysis predicting self-efficacy (SGSES) from CFQ and PSS. Both cognitive failures (B = -0.170, *p* = 0.001) and stress (B = -0.483, *p* = 0.005) significantly reduced self-efficacy, explaining 7.9% of its variance (adjusted R² = 0.079). The model was statistically significant (F = 12.435, *p* < 0.001).

**Table 5:** A multiple regression analysis between self-efficacy, stress, and cognitive lapses among the participants (*n* = 268)

Model	B	S.E.	t	*P*
CFQ	-0.170	0.048	-3.522*	0.001*
PSS	-0.483	0.170	-2.833*	0.005*
Constant	82.323	4.634	17.765*	< 0.001*

R^2^ = 0.086 Adjusted R^2^ = 0.079 F = 12.435* *Significant at *P* ≤ 0.05

S.E: Standard Error; B: Standardized Coefficients

CFQ: Cognitive Failure Questionnaire

PSS: Perceived Stress Scale

SGSES: Sherer’s General Self-Efficacy Scale

**Fig. 3:**
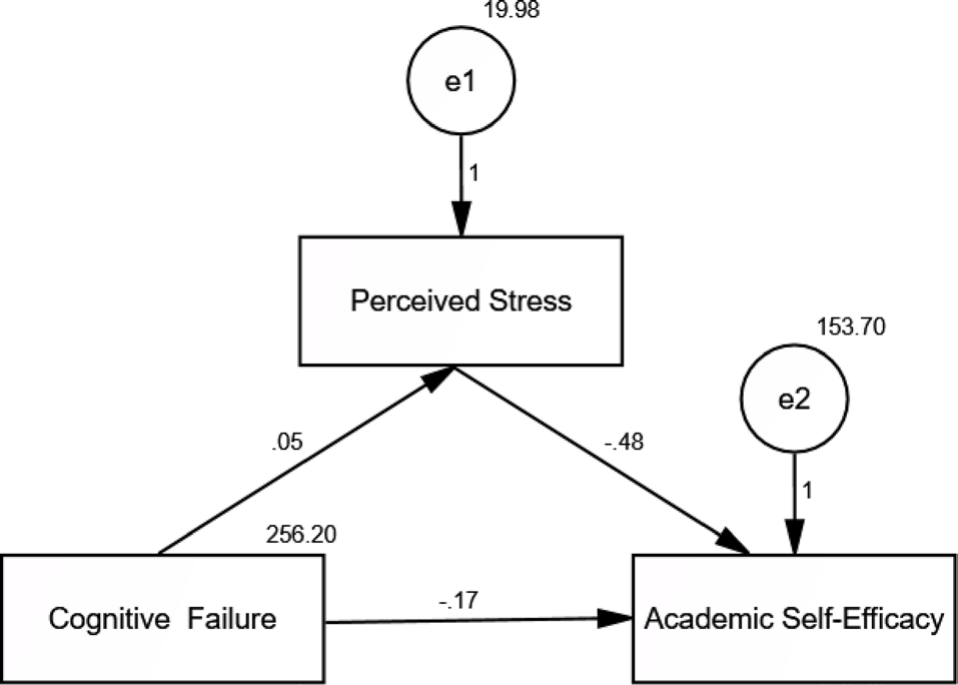
Path analysis to detect the direct and indirect effects of perceived stress on academic self-efficacy, mediated by cognitive failure

Table 6 and Fig. 3 outline path analysis results testing cognitive failure as a mediator between perceived stress and academic self-efficacy. Stress had a direct negative effect on self-efficacy (β = -0.483, *p* = 0.004), while cognitive failure partially mediated this relationship (indirect effect = -0.025, CR = -3.536, *p* < 0.001). The model fit was acceptable (CFI = 0.918, RMSEA = 0.093), with a significant overall χ² (*p* < 0.001).

**Table 6:** Path analysis to detect the direct and indirect effects of perceived stress on academic self-efficacy, mediated by cognitive failure

Variable 1		Variable 2	Direct effect	Indirect effect	CR	*p*-value
Perceived Stress	←	Cognitive Failure	0.052	—	3.014*	0.003*
Academic Self-Efficacy	←	Cognitive Failure	–0.170	-0.025	-3.536*	< 0.001*
Academic Self-Efficacy	←	Perceived Stress	–0.483		-2.844*	0.004*

CFI = Comparative Fit Index; IFI = Incremental Fit Index; RMSEA = Root Mean Square Error of Approximation

Model fit parameters: CFI = 0.918; IFI = 0.936; RMSEA = 0.093

Model χ² = 10.961* (*p* < 0.001*)

## Discussion

Graduate education is essential for professional advancement, career development, personal growth, and economic progress. However, it is often accompanied by significant stress, which can negatively impact candidates by impairing their coping skills, academic performance, and cognitive functions, ultimately hindering success [19, 35, 36]. Cognitive failure negatively impacts one’s quality of life, as mistakes can have serious and life-threatening implications [26, 37]. Experiencing distractions, time constraints, or chaos can lead to blunders [28]. Self-efficacy, a key motivational and cognitive factor, promotes engagement, persistence, self-regulation, and effective stress management [36, 38]. This study examined the interplay between perceived stress, cognitive failure, and academic self-efficacy among postgraduate nursing students.

The findings of this study revealed that cognitive failure serves as a partial mediator in the relationship between stress and self-efficacy. Stress directly increases cognitive failure, which in turn significantly reduces self-efficacy. The indirect pathway was stronger than the direct effect of stress on self-efficacy, supporting a partial mediation model. These results align with Lazarus’s (1984) cognitive stress theory, which posits that stress impairs cognitive function, leading to a diminished sense of self-belief in academic capabilities [33].

The positive correlation between stress and cognitive failure was one of the key findings of the study. These findings highlight that stress undermines students’ academic confidence both directly and indirectly by disrupting their cognitive processes. These results align with the theoretical proposition that stress negatively affects attention and working memory, key aspects of cognitive function essential for successful learning and clinical decision-making [2, 11, 13]. They also support previous findings by El-Kasaby (2024), who showed that academic emotions can significantly predict cognitive failure, further reinforcing the role of internal psychological states in shaping cognitive outcomes [39]. The study explained that 85% of the variation in cognitive failure and positive emotions was more predictive than negative emotions. These findings underscore the importance of promoting positive academic experiences to mitigate cognitive decline.

Notably, the indirect path from stress to self-efficacy via cognitive failure emerged as a critical pathway, suggesting that interventions aimed at reducing cognitive failure, such as cognitive training or mindfulness, may indirectly enhance students’ self-belief. This complements prior findings by Fürtjes et al. (2023), who conducted a series of analyses using multiple linear regressions and hierarchical linear modeling to investigate micro- and macro-level relationships [40]. At the micro-level, the results indicated that self-efficacy serves to mitigate the influence of stress on depression; however, this effect did not extend to anxiety in daily contexts, with no evidence of longitudinal implications. Conversely, at the macro level, self-efficacy was found to mitigate the impacts of stress on anxiety, yet this protective factor did not apply to depression. In terms of longitudinal findings, significant direct effects were observed for self-efficacy with anxiety, as well as stress concerning both depression and anxiety. The strong influence of stress on depression appears resistant to mitigation by self-efficacy at a broad level. In contrast, the protective impact of self-efficacy on anxiety may not consistently apply in daily contexts. These findings suggest that depression treatments should prioritize stress reduction, whereas anxiety therapies might benefit from strengthening self-efficacy to address avoidant behaviors.

Moreover, the study revealed that as cognitive failures increase, academic self-efficacy decreases, suggesting that students who frequently experience attention lapses or memory errors perceive themselves as less academically competent. This relationship was clearly demonstrated in the regression model, where cognitive failure had a stronger predictive effect on self-efficacy than stress. These findings could be explained in a way that graduate candidates often juggle multiple responsibilities, such as being a student, partner, caregiver, and usually carry financial burdens. Managing these roles can be overwhelming, which increases stress and negatively impacts cognitive performance. Stress can lead to low self-esteem and an increase in depressive symptoms. Strategies such as effective time management, seeking support from peers or mentors, and prioritizing self-care are crucial for navigating this. Similarly, Stillwell et al. (2017) reported that perceived stress among graduate nursing candidates has been widely recognized for decades, with familiar sources including financial challenges, time management issues, role responsibilities, relationships, competing obligations, academic demands, and clinical practicum experiences involving client interactions [41]. Pluviano et al. (2016) supported the idea that stress is linked to both subjective and objective prospective memory problems, as well as more severe daily cognitive symptoms [42]. In addition, compared to participants who experienced less stress, high-stress individuals showed less resilience and complained more about psychological symptoms (such as anxiety and despair). These findings align with the study by Santangelo et al. (2021), which analyzed data from 3605 non-healthcare workers and 570 healthcare professionals, including doctors, nurses, psychologists, lab technicians, and medical waste handlers [43]. The study found that 27.7% of non-healthcare workers and 25.9% of healthcare professionals reported experiencing cognitive failure, with no statistically significant difference between the groups (χ² = 0.94, *p* = 0.33). Participants were further categorized into employment groups: 2377 were unemployed, 1285 were classified as “smart workers,” and 513 were “non-smart workers.” Notably, “non-smart workers” (26.5%) reported fewer cognitive failures than unemployed individuals (28.2%). Research suggests that cognitive failures are linked to stress, anxiety, and poor academic performance. Students often face stressful conditions, struggle with time management and procrastination, feel pressured to achieve success, and experience anxiety related to studying and exams [27]. Alwawi et al. (2024) confirmed that cognitive distortions linked to stress and anxiety significantly impact mental health. This study examined the prevalence of these distortions among nursing students at a university in Palestine, revealing that 5% had severe levels, 33% had moderate levels, 47% had mild levels, and 15% had healthy levels. The most common distortions were emotional reasoning, perfectionist thinking, and “What if?” questions, while polarized thinking and overgeneralization were the least frequent. Single, first-year, and younger students exhibited higher levels of cognitive distortions [44]. These findings underscore the importance of recognizing and addressing cognitive distortions to support the mental health of nursing students, highlighting the need for preventive services in universities. Additionally, Munoz et al. (2015) discovered links between exposure to stress events and adult cognitive functions [45]. Loss of cognitive function is associated with higher reports of life events or ongoing obstacles among persons with pre-existing cognitive impairment (i.e., those who initially exhibited indicators of moderate cognitive impairment. Notwithstanding the conflicting findings of studies on life events and cognitive functions, the detrimental effects of these occurrences on functioning appear to vary depending on the type of stressor and the perceived consequences of the event for an individual’s well-being. This implies that even exposure alone is not a reliable indicator of declining cognitive functions.

Interestingly, the findings showed that the indirect pathway was stronger than the direct effect of stress on self-efficacy, supporting a partial mediation model. This may reflect individual differences in stress appraisal and coping, as suggested by Lazarus’s (1984) cognitive stress theory. Students with higher self-efficacy may interpret stressors as challenges rather than threats, thus preserving their academic confidence despite external pressures [33]. Individuals with high self-efficacy are more inclined to perceive challenges, enhancing their ability to manage stress effectively. Self-efficacy mediates the relationship between stressors and psychological stress by influencing how demands are appraised and the coping strategies used. Academic self-efficacy fully mediates the relationship between stress and these evaluations. Additionally, physiological arousal linked to stress and anxiety impacts self-efficacy judgments, which heightened stress and anxiety levels can diminish [46]. In a cohort of students enrolled in the Master of Science in Nursing program, several prominent themes were identified: workload, research activities, task prioritization, insufficient support, inadequate feedback, balancing academic and personal life, elevated stress levels, and expectations imposed by faculty members. The participants conveyed that their experiences in graduate education were characterized by significant stress and a sense of being overwhelmed. This perceived stress was primarily linked to a stringent curriculum and various contributing factors, including substantial workloads, time constraints, inadequate support systems, protracted response times from research advisors, and delays in feedback from the Ethics Review Committee for their research proposals [19].

Moreover, socio-demographic variables influenced outcomes in specific ways. The current study findings indicated that age did not significantly impact the relationship between socio-demographic characteristics, cognitive failure, perceived stress, and self-efficacy. Gender differences were observed, with females reporting higher cognitive failure scores than males, although perceived stress levels were similar between the two groups. Marital status had a significant influence on all measures, with singles recording the highest scores across all measures. Education level did not considerably affect CFQ or PSS scores between groups, though technical institute graduates reported higher perceived stress. These results highlight the significant impact of gender, marital status, and education on shaping psychological outcomes. In this regard, Kumareswaran et al. (2023) stated that cognitive capacity is influenced by various aspects, including socio-demographics, lifestyle, familial situations, and social networks [26]. Social support refers to the aid that individuals provide within a person’s social network. The level of support varies based on the number, intensity, and frequency of social interactions. Pluviano et al.‘s (2016) hierarchical regression model showed that, in addition to stress, age, and individual differences in negative symptoms, memory strategy use also explained variance in prospective memory errors [42]. Additionally, these latter factors mediated the association between stress and future memory. These results underscore the potential significance of individual characteristics in mitigating the adverse effects of daily stress and support the notion that stress may significantly impair future memory functioning. The study by Goel et al. (2016) examined the impact of stress on self-efficacy and emotional intelligence among college students, with a particular focus on comparing humanities and science students across genders [47]. The hypothesis, rooted in the societal belief that females experiencing higher levels of stress would exhibit lower self-efficacy and emotional intelligence than their male counterparts, was not entirely supported by the findings. While female science students with elevated stress levels demonstrated lower self-efficacy, they outperformed males in managing their emotions. Therefore, the hypothesis was partially validated: although higher stress levels impair females’ capabilities more than males’, they display superior emotional regulation skills compared to their male peers.

### Strengths and limitations

The study highlights essential correlations between this population’s cognitive failure, perceived stress, and self-efficacy. However, the study’s cross-sectional design limits causal inferences regarding these relationships, and reliance on self-reported measures may introduce bias due to social desirability or lack of self-awareness. Additionally, conducting the study in a controlled environment may not fully capture real-world complexities, potentially limiting ecological validity. Another limitation is that the sample may lack demographic diversity, which could affect the generalizability of the findings across different nursing student populations. Lastly, this study did not account for potentially confounding factors, such as sleep quality and social support, which may have influenced the observed results. Future research should address these limitations by employing longitudinal designs, seeking a more diverse participant pool, and considering the inclusion of the above confounding factors.

### Conclusion and recommendation

This study revealed that cognitive failure significantly mediates the relationship between perceived stress and academic self-efficacy among postgraduate nursing students. The path analysis demonstrated that perceived stress directly increases cognitive failure, which in turn strongly diminishes self-efficacy. Although the direct impact of stress on self-efficacy was marginally significant, the indirect pathway through cognitive failure was notably more substantial, supporting a partial mediation model. These findings highlight the crucial role of cognitive functioning as a mechanism through which stress undermines students’ confidence in their academic abilities. Moreover, cognitive failure had a more pronounced effect on self-efficacy than stress alone, suggesting that interventions aimed at reducing cognitive slips, such as attention training or cognitive restructuring, may help enhance academic confidence. Gender and marital status were also significant, with female and single students reporting higher levels of cognitive failure and perceived stress.

### Nursing implications

The current study carries significant implications for nursing education and practice. Elucidating the correlation between stress and cognitive failure can facilitate the establishment of more effective support systems within nursing education. Educational institutions may consider implementing comprehensive stress management initiatives, counseling services, and wellness programs designed explicitly for postgraduate nursing students, including mindfulness training, cognitive-behavioral interventions, and wellness initiatives tailored to this population. Moreover, this study’s findings can help inform the development of curricula that are aware of the stressors encountered by postgraduate nursing students. This entails establishing a balance between theoretical instruction and practical application, while ensuring the curriculum enhances academic self-efficacy. Furthermore, the insights derived from this research can serve as a foundation for policy reform within educational institutions. Policies that advocate a balanced academic life ensure sufficient study breaks and provide flexible scheduling options that alleviate stress and avert burnout.

## Data Availability

The datasets used and analyzed during the current study are available from the corresponding author upon reasonable request.

## References

[1] Bhurtun HD, Azimirad M, Saaranen T, Turunen H. Stress and coping among nursing students during clinical training: an integrative review. J Nurs Educ. 2019;58(5):266–72. 31039260 10.3928/01484834-20190422-04

[2] Yang D, Zheng W, Li N, Wang X, Chen W, Liu Z, et al. The mediating role of psychological capital on the relationship between perceived stress and self-directed learning ability in nursing students. BMC Nurs. 2024;23(1):404. 38886795 10.1186/s12912-024-02094-6 PMC11181674

[3] El-Sayed MM, Abd El-Fatah Abd-Elhamid E, Abd El-Gawad Mousa M. Academic, Motivation. Academic self-efficacy and perceived social support among undergraduate nursing students, Alexandria University, Egypt. Assi Sci Nurs J. 2021;9(24).

[4] El-Ashry AM, Elhay ESA, Taha SM, Salem ESAEHES, El-Sayed MM. Impact of virtual group-based acceptance and commitment therapy on social adjustment and work-family conflict among intern nurses: a randomized control trial. BMC Psychiatry. 2023;23(1). 10.1186/s12888-023-05045-8 PMC10391863 37525125

[5] El-Ashry AM, Taha SM, Elhay ESA, Hammad HAH, Khedr MA, El-Sayed MM. Prevalence of imposter syndrome and its association with depression, stress, and anxiety among nursing students: a multi-center cross-sectional study. BMC Nurs. 2024;23(1). 10.1186/s12912-024-02414-w PMC11603883 39605033

[6] El-Sayed MM, AbdElhay ES, Hawash MM, Taha SM. The power of laughter: a study on humor and creativity in undergraduate nursing education in Egypt. BMC Nurs. 2024;23(1). 10.1186/s12912-024-01913-0 PMC11034109 38649907

[7] Labrague LJ, McEnroe-Petitte DM, Al Amri M, Fronda DC, Obeidat AA. An integrative review on coping skills in nursing students: implications for policymaking. Int Nurs Rev. 2018;65(2):279–91. 28664984 10.1111/inr.12393

[8] El-Sayed MM, Ghazi GA, Kamal MA, Khedr MA. Investigating fear, depressive symptoms, and coping mechanisms among Egyptian nursing students amidst the COVID-19 pandemic: A cross-sectional study. BMC Nurs. 2024;23(1):461. 38978016 10.1186/s12912-024-02104-7 PMC11229188

[9] El-Sayed MM, Elhay ESA, Hawash MM, Sonbol HM, Taha SM. A closer look: obsessive-compulsive symptoms among intern nurses amidst COVID-19 pandemic. BMC Nurs. 2024;23(1):214. 38549136 10.1186/s12912-024-01872-6 PMC10976757

[10] Chaabane S, Chaabna K, Bhagat S, Abraham A, Doraiswamy S, Mamtani R et al. Perceived stress, stressors, and coping strategies among nursing students in the middle East and North africa: an overview of systematic reviews. Syst Rev. 2021;10(1). 10.1186/s13643-021-01691-9 PMC8101235 33952346

[11] Albaqawi H, Albagawi B, Butcon V, Alsaqri S, Pangket P. Level of perceived stress and coping styles through positive mental health among nursing students in hail, Saudi Arabia. Stress. 2022;27:40.

[12] Ngoc NB, Tuan N, Van. Stress among nursing students in vietnam: prevalence and associated factors. Int Nurs Rev. 2024;71(1):28–34. 36696254 10.1111/inr.12831

[13] Yang J, Herawati N, Namjoo F. Emotional clarity and stress vulnerability as predictors of cognitive failures. J Personality Psychosom Res (JPPR). 2024;2(3):4–10.

[14] Mohamed NA, Ali SO, Ehrahim EEE, Ahmed AL, Wahba AM. Predictors of academic and clinical stress among nursing students. SAGE Open Nurs. 2024;10:23779608241290390. 10.1177/23779608241290392 PMC11514110 39469726

[15] Carrigan N, Barkus E. A systematic review of cognitive failures in daily life: Healthy populations. 2016. 10.1016/j.neubiorev.2016.01.010 26835660

[16] Hall LM, FERGUSON-PARÉ M, Peter E, White D, Besner J, Chisholm A, et al. Going blank: factors contributing to interruptions to nurses’ work and related outcomes. J Nurs Manag. 2010;18(8):1040–7. 21073575 10.1111/j.1365-2834.2010.01166.x

[17] Smith JG, Urban RW, Wilson ST. Association of stress, resilience, and nursing student incivility during COVID-19. Nursing forum. Wiley Online Library; 2022. pp. 374–81. 10.1111/nuf.12694 35032050

[18] Yoo HJ, Marshall DT. Exploring graduate students’ perceived helplessness, self-efficacy, social support, and satisfaction. Stud Graduate Postdoctoral Educ. 2025;16(1):73–89.

[19] El-Sayed MM, Taha SM, AbdElhay ES, Hawash MM. Understanding the relationship of academic motivation and social support in graduate nursing education in Egypt. BMC Nurs. 2024;23(1):12. 38166845 10.1186/s12912-023-01671-5 PMC10759519

[20] Fard FS, Asayesh H, Hosseini MHM, Sepahvandi M. Motivation, self-efficacy, stress, and academic performance correlation with academic burnout among nursing students. J Nurs Midwifery Sci. 2020;7(2):88–93.

[21] Okasha T, Shaker N, El-Gabry DA, Burnout. Mental Health, and Wellbeing among egyptian students. The mental health of medical students: supporting wellbeing in medical education. 2024;120.

[22] Cevallos M, Egger M. STROBE (Strengthening the Reporting of Observational studies in Epidemiology). Guidelines for reporting health research: a user’s manual. 2014;169–79.

[23] Charan J, Kaur R, Bhardwaj P, Singh K, Ambwani SR, Misra S. Sample size calculation in medical research: a primer. Ann Natl Acad Med Sci. 2021;57(02):74–80.

[24] El-Ashry AM, El-Sayed MM, Elhay ESA, Taha SM, Atta MHR, Hammad HAH, et al. Hooked on technology: examining the co-occurrence of nomophobia and impulsive sensation seeking among nursing students. BMC Nurs. 2024;23(1):18. 38166837 10.1186/s12912-023-01683-1 PMC10763039

[25] Broadbent DE, Cooper PF, FitzGerald P, Parkes KR. The cognitive failures questionnaire (CFQ) and its correlates. Br J Clin Psychol. 1982;21(1):1–16. 7126941 10.1111/j.2044-8260.1982.tb01421.x

[26] Kumareswaran S, Muhadi U, Farhan A, Sathasivam J. Relationship between sociodemographic factors and cognitive failures among employees. Eur J Humanit Social Sci. 2023;3(1):16–22.

[27] Dzubur A, Koso-Drljevic M, Lisica D. Understanding cognitive failures through psychosocial variables in daily life of students. J Evol Med Dent Sci. 2020;9(45):3382–6.

[28] Arnetz JE, Arble E, Sudan S, Arnetz BB. Workplace cognitive failure among nurses during the COVID-19 pandemic: an international. J Environ Res Public Health. 2021;18(19):10394. 10.3390/ijerph181910394 PMC8508323 34639695

[29] Cohen S. Perceived stress in a probability sample of the United States. The social psychology of health/Sage. 1988.

[30] Baik SH, Fox RS, Mills SD, Roesch SC, Sadler GR, Klonoff EA, et al. Reliability and validity of the perceived stress Scale-10 in Hispanic Americans with english or Spanish Language preference. J Health Psychol. 2019;24(5):628–39. 28810432 10.1177/1359105316684938 PMC6261792

[31] Chaaya M, Osman H, Naassan G, Mahfoud Z. Validation of the Arabic version of the Cohen perceived stress scale (PSS-10) among pregnant and postpartum women. BMC Psychiatry. 2010;10:1–7. 21159169 10.1186/1471-244X-10-111 PMC3016315

[32] Sherer M. The self-efficacy scale: construction and validation. University of Alabama; 1982.

[33] Lazarus RS. Stress, appraisal, and coping. Volume 464. Springer; 1984.

[34] Aldrich JO. Using IBM SPSS statistics: an interactive, hands-on approach. Sage; 2018.

[35] Duhamel KV. Bringing Us back to our creative senses: fostering creativity in graduate-level nursing education: A literary review. Nurse Educ Today. 2016;45:514. 10.1016/j.nedt.2016.06.016 27429404

[36] Yoo HJ, Marshall DT. Exploring graduate students’ perceived helplessness, self-efficacy, social support, and satisfaction. Studies in Graduate and Postdoctoral Education. 2024.

[37] Lewis LS. Community College Nursing Students’ Experience of Repeating a Course After Academic Failure. 2016.

[38] Karwowski M. The dynamics of creative self-concept: changes and reciprocal relations between creative self-efficacy and creative personal identity. Creat Res J. 2016;28(1):99–104.

[39] El-Kasaby WH. The relative contribution of positive and negative academic emotions in predicting cognitive failure in university students. Contemp Readings Law Social Justice. 2024;16(1):1632–46.

[40] Fürtjes S, Voss C, Rückert F, Peschel SKV, Kische H, Ollmann TM, et al. Self-efficacy, stress, and symptoms of depression and anxiety in adolescents: an epidemiological cohort study with ecological momentary assessment. J Mood Anxiety Disorders. 2023;4:100039. 10.1016/j.xjmad.2023.100039 PMC12243988 40656967

[41] Stillwell SB, Vermeesch AL, Scott JG. Interventions to reduce perceived stress among graduate students: A systematic review with implications for evidence-based practice. Worldviews Evid Based Nurs. 2017;14(6):507–13. 28795775 10.1111/wvn.12250

[42] Pluviano S, Gamboz N, Brandimonte MA. On the effect of stress on cognitive failures in everyday life: A look into prospective memory errors. In: Poster presented at the 2nd International Meeting of the Psychonomic Society, Granada, Spain. 2016.

[43] Santangelo G, Baldassarre I, Barbaro A, Cavallo ND, Cropano M, Maggi G, et al. Subjective cognitive failures and their psychological correlates in a large Italian sample during quarantine/self-isolation for COVID-19. Neurol Sci. 2021;42(7):2625–35. 33914195 10.1007/s10072-021-05268-1 PMC8082482

[44] Alwawi A, Alsaqqa HH. Protecting the mental health of the future workforce: exploring the prevalence of cognitive distortions among nursing students. Nurs Manage. 2024;31(5). 10.7748/nm.2023.e2077 36891681

[45] Munoz E, Sliwinski MJ, Scott SB, Hofer S. Global perceived stress predicts cognitive change among older adults. Psychol Aging. 2015;30(3):487. 26121285 10.1037/pag0000036 PMC4559145

[46] Zajacova A, Lynch SM, Espenshade TJ. Self-efficacy, stress, and academic success in college. Res High Educ. 2005;46:677–706.

[47] Goel A, Bardhan S. Effect of stress on self-efficacy and emotional intelligence among college students of humanities and sciences: A study on gender differences. Int J Appl Res. 2016;2(12):318–28.

